# Differential Cadmium Distribution and Translocation in Roots and Shoots Related to Hyper-Tolerance between Tall Fescue and Kentucky Bluegrass

**DOI:** 10.3389/fpls.2017.00113

**Published:** 2017-02-03

**Authors:** Qin Dong, PeiXian Xu, ZhaoLong Wang

**Affiliations:** ^1^School of Agriculture and Biology, Shanghai Jiao Tong UniversityShanghai, China; ^2^Shanghai Administration Department of Green CityShanghai, China

**Keywords:** cadmium, fluorescence, cellular distribution, translocation, hyper-tolerance, tall fescue, Kentucky bluegrass

## Abstract

Phytoremediation efficiency mainly depends upon mechanisms in the uptake and translocation of soil contaminants. Cadmium (Cd) distribution and translocation in roots and shoots of tall fescue (*Festuca arundinacea*) and Kentucky bluegrass (*Poa pratensis*) were observed using fluorescence spectroscopy with a laser confocal scanning microscope. No difference in root Cd accumulations was detected between these two turfgrass species. Kentucky bluegrass transported more Cd into the stele for root-to-shoot translocation and resulted in significantly higher Cd concentration in leaves. In tall fescue, less Cd was transported into the stele in roots and more Cd was excreted to the cuticle layer in leaves. These results suggested that both turfgrass species were hypertolerant to Cd through distinct distribution patterns in leaves and roots.

## Introduction

Cadmium (Cd) pollution is one of the most serious environmental problems and there were approximately 20 million hectares of cultivated lands contaminated by Cd (Yu et al., 2006; Xue et al., 2014; Liu et al., 2015). Phytoremediation is a cost-effective and environment-friendly remediation technique for soil Cd contamination (Salt et al., 1998; Wan et al., 2016). Plants can absorb, translocate, sequestrate, degradate and extract contaminants from the contaminated soil without any damage of soil physical and chemical properties. After that, plants can be harvested, and processed by drying, ashing or composting. Some metals can be reclaimed from the ash (Raskin et al., 1997). The efficiency of phytoremediation mainly depends upon the capacities of plants (hyperaccumulators) to take up the heavy metal and remove from the soil (Bell et al., 2014). The plant extraction and tolerance to Cd involve many processes, which include Cd movement and availability in the soil, Cd absorption and accumulation in root tissues, Cd translocation from roots to the above-ground shoots via the vascular system, Cd detoxification and deposit in shoot tissues (Lux et al., 2011; Zhou et al., 2015; Cornu et al., 2016).

Cadmium is very toxic to plants and the Cd-tolerant threshold in most reported hyperaccumulators were below 25 mg kg^-1^ of soil (Wei et al., 2004, 2008, 2009). Our pervious study showed that tall fescue (*Festuca arundinacea*) and Kentucky bluegrass (*Poa pratensis*) could tolerate up to 200 and 100 mg kg^-1^ of soil Cd concentration, respectively, without any significant decline of turf quality (Xu et al., 2014). Plants of tall fescue and Kentucky bluegrass accumulated 44.5- and 40.8-fold more Cd than Cd hyperaccumulator, *Solanum nigrum*, without any toxic symptoms (Xu and Wang, 2014). These results indicated that tall fescue and Kentucky bluegrass could be good candidate plants for phytoextraction or phytostabilization of Cd contaminated soils. However, it is still unclear about the mechanism of Cd hypertolerance in these two turfgrass species.

Cadmium distribution in plants tissues and cells is important for understanding the mechanism of hypertolerance. Therefore, the objectives of this study were (i) to observe *in situ* Cd distribution and translocation in roots and leaf tissues, (ii) interpret the differences of Cd hypertolerance between tall fescue and Kentucky bluegrass.

## Materials and Methods

Tall fescue (cv. Barlexas) and Kentucky bluegrass (cv. Midnight) were planted in sand-based fields. Mature plants were transplanted into a hydroponic culture system in this study. After roots and old leaves were removed, plants were transplanted into 5 L plastic pots containing 1/2 strength Hoagland’s nutrient solution (Hoagland and Arnon, 1950) with aeration to induce new root development in growth chambers.

The growth chambers used in this experiment were constructed under the Chinese National Standard (GB/T 32710.6-2016 and GB/T 32710.7-2016). There were13 sensors (temperature, irradiation, humidity, CO_2_) evenly distributed in the 3 m × 3 m growth chamber to monitor the environmental variables in the growth chamber. All environmental parameters were controlled by the artificial intelligence programs and were calibrated each month. During the whole experimental period, the variations of all monitored environmental parameters in the growth chamber were controlled ≤5% at the canopy level. In the experiment, canopy temperature was controlled at 25/20 ± 2°C (day/night). Canopy photosynthetically active radiation was controlled at 400 μmol m^-2^s^-1^ with 14-h photoperiod. Relative humidity was controlled at 75 ± 2%.

After new roots were developed and reached above 10 cm long, plants were transferred to the modified 1/2 strength Hoagland’s nutrient solution containing 0.3 mM Cd^2+^ supplied in the form of CdCl_2_. The control group was maintained under the same environmental conditions, except without the addition of CdCl_2_. All treatments and controls were replicated four times in the experiment. The nutrient solution was renewed every week and the pH was adjusted to 6.5 with 0.1 M HCl or 0.1 M NaOH.

Cadmium fluorescence observation was conducted according to Xiong et al. (2009). After 7 days of Cd treatment, fresh roots and the first fully expanded leaves were sampled for cross section with freezing microtome. All sections were 40 μm thick and were stained with the assay reagent from the LEADMIUM kit (Invitrogen) for 60 min in the dark and then washed three times with assay buffer for 5 min each time. Samples were observed with a laser confocal scanning microscope (Leica TCS SP5-II) with excitation and emission wavelengths of 488 and 520 nm, respectively.

After 60 days of Cd treatment, plants were harvested and separated into roots and shoots. The roots were immersed in 20 mM EDTA-Na_2_ solution for 15 min to remove Cd that was adhered to the root surface (Xu and Wang, 2013), washed with deionized water and then dried with an absorbent paper. The roots and shoots were dried at 100°C for 10 min and 80°C to a constant weight in an oven. Dry samples were ground, passed through a 100-mesh (0.15 mm) sieve and digested in supra-pure concentrated HNO_3_ and HClO_4_ (4:1, v/v) at 130–150°C and diluted to 100 mL. The concentration of Cd was determined using inductively coupled plasma-atomic emission spectroscopy (ICP-AES, iCAP6300, Thermo, USA).

The hydroponic experiment was repeated three times in a 2-years period. A histochemical method was used for Cd detection in the first experiment (**Supplementary Data sheet 1**) according to Seregin and Kozhevnikova (2011). The photos presented in the paper were from Cd fluorescence method (Xiong et al., 2009) in the second experiment, which showed more clear Cd distribution in plant tissues. The third repeated experiment was conducted for observation of the pathway of leaf excretion. The roots and shoots were harvested to determine the Cd concentrations in all three repeated experiments and the repeated experiments showed the same results. The Cd data presented in this paper was from the same samples used for Cd fluorescence observation in the second repeated experiment.

All data are presented as means of four replicates. Statistical analysis were performed with the software SAS (version 9.1, SAS Institute Inc., Cary, NC, USA) using the general linear model (GLM) procedure. Least significance difference (LSD) at a 0.05 probability level was used to detect the differences between treatment means.

## Results

**FIGURE 1:**
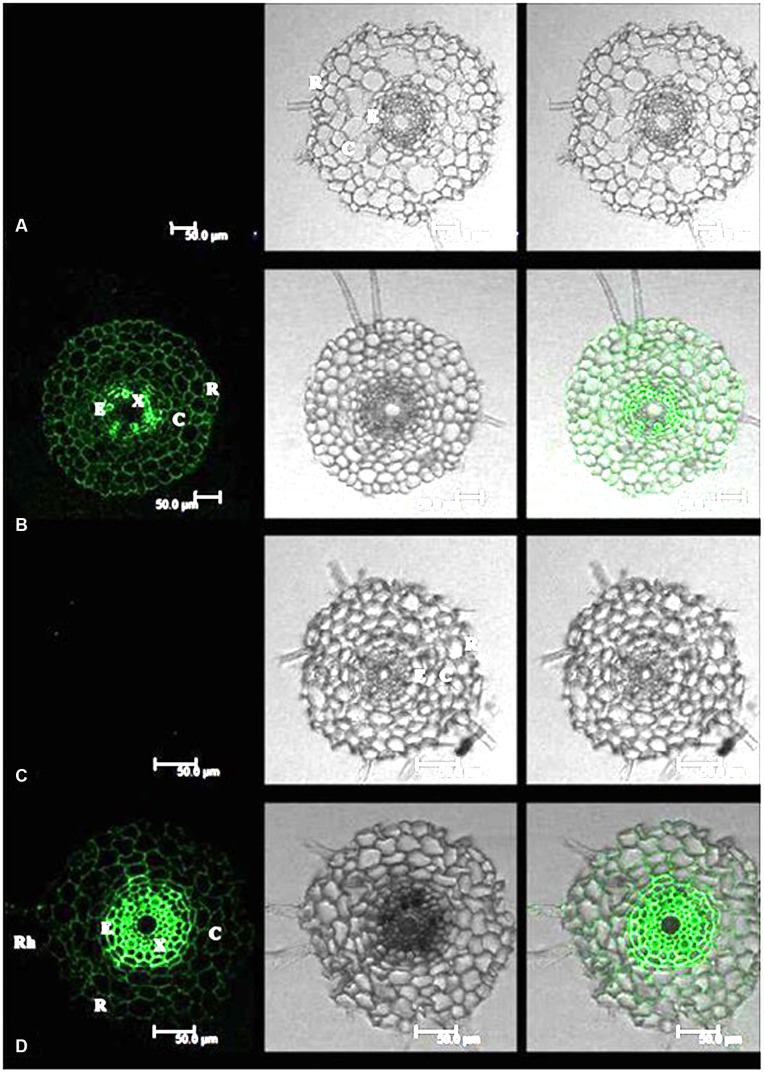
**Cadmium (Cd) distributions in the root tissues of tall fescue **(A,B)** and Kentucky bluegrass **(C,D)**.** **(A)** Tall fescue control; **(B)** tall fescue Cd treatment under 0.3 mM Cd^2+^ for 7 days; **(C)** Kentucky bluegrass control; **(D)** Kentucky bluegrass Cd treatment under 0.3 mM Cd^2+^ for 7 days. R, rhizodermis; C, cortex; E, endodermis; X, xylem. Left column: Cd fluorescence image; Middle column: root transverse sectional image; Right column: combined image with Cd fluorescence and root section.

Cadmium fluorescence was clearly observed in the epidermis, cortex and stele of roots in both tall fescue and Kentucky bluegrass (**Figure 1**). No fluorescence was observed in control plants, indicating that the fluorescence spectroscopy is a specific reliable method for Cd localization in plant tissues. In roots of tall fescue, Cd fluorescence showed even distribution in the epidermis, cortex, and stele; the strongest Cd fluorescence was observed in xylem vessels in the stele (**Figure 1B**). Cd fluorescence in root tissues of Kentucky bluegrass showed an uneven distribution, with much stronger Cd fluorescence in the stele and less fluorescence in the epidermis and cortex (**Figure 1D**).

**FIGURE 2:**
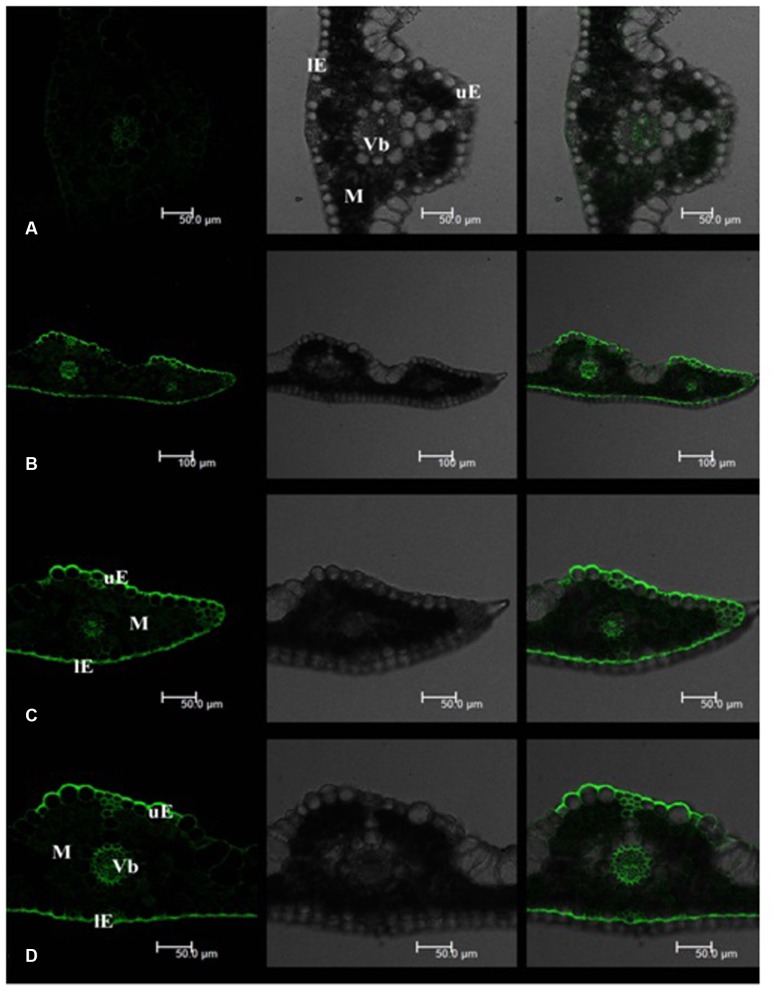
**Cadmium distributions in the leaf tissues of tall fescue.** **(A)** Tall fescue control; **(B–D)** tall fescue Cd treatment under 0.3 mM Cd^2+^ for 7 days. uE, upper epidermis; lE, lower epidermis; M, mesophyll; Vb, vascular bundle. Left column: Cd fluorescence image; Middle column: leaf transverse sectional image; Right column: combined image with Cd fluorescence and leaf section.

**FIGURE 3:**
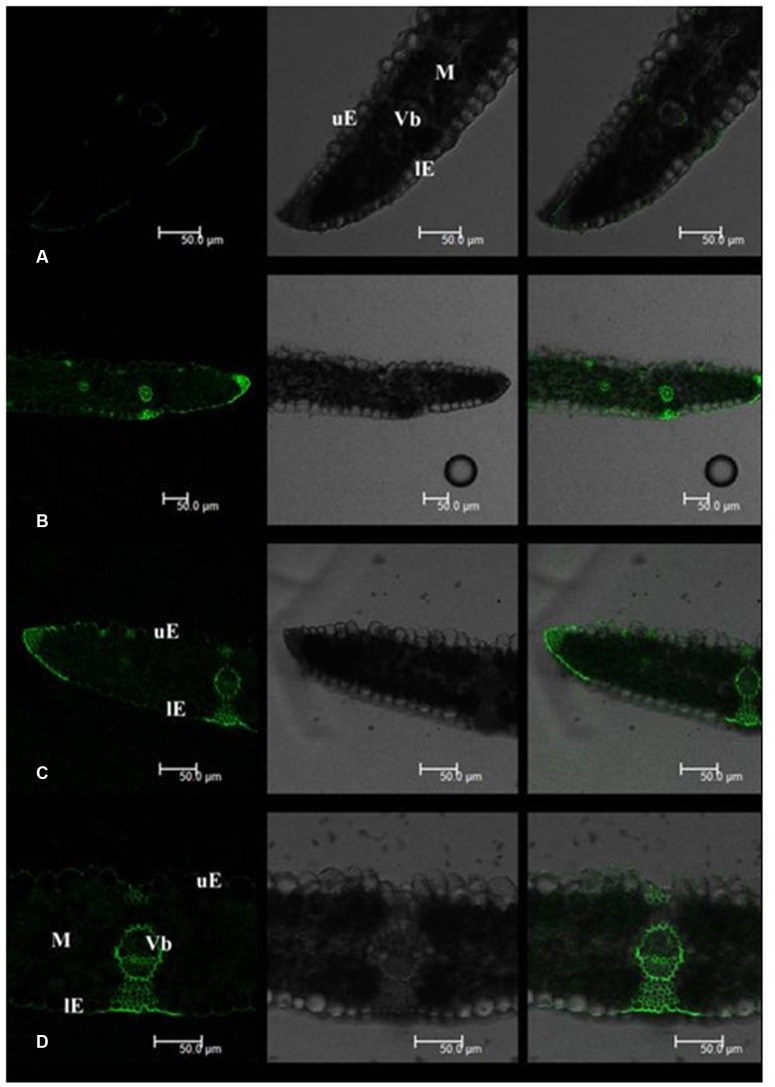
**Cadmium distributions in the leaf tissues of Kentucky bluegrass.** **(A)** Kentucky bluegrass control; **(B–D)** Kentucky bluegrass Cd treatment under 0.3 mM Cd^2+^ for 7 days. uE, upper epidermis; lE, lower epidermis; M, mesophyll; Vb, vascular bundle. Left column: Cd fluorescence image; Middle column: leaf transverse sectional image; Right column: combined image with Cd fluorescence and leaf section.

Leaf Cd fluorescence showed uneven patterns in both tall fescue and Kentucky bluegrass (**Figures 2** and **3**). In tall fescue leaves, the strongest Cd fluorescence was observed in vascular bundles and the epidermis. Mesophyll tissues showed the weakest Cd fluorescence (**Figure 2**). There was very strong Cd fluorescence in the cuticle layer of epidermis all around the leaf surface in tall fescue. In Kentucky bluegrass leaves, the strongest Cd fluorescence was detected in veins and leaf margins (**Figure 3**). There was much weaker Cd fluorescence in the epidermis between veins than that within the vein and leaf margins. Mesophyll tissues showed stronger Cd fluorescence in Kentucky bluegrass compared with those in tall fescue.

**FIGURE 4:**
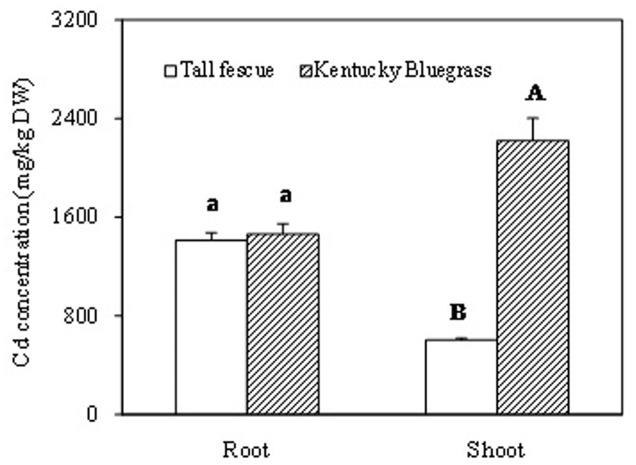
**The Cadmium accumulation in root and shoot of tall fescue and Kentucky bluegrass after 60 days of Cd treatment. ‘A’ and ‘B’ represent the significant difference of Cd concentrations between the shoots of tall fescue and Kentucky bluegrass.** ‘a’ represents no significant difference of Cd concentrations between the roots of tall fescue and Kentucky bluegrass.

After 60 days of Cd treatment, root Cd concentration showed no significant difference between tall fescue and Kentucky bluegrass (**Figure 4**). However, shoot Cd concentration in Kentucky bluegrass was significantly higher than that in tall fescue. Kentucky bluegrass shoot accumulated about 3.7-fold higher amount of Cd than tall fescue.

## Discussion

Cadmium can be absorbed into the rhizodermis and cortex of roots either via apoplastic or symplastic pathways (Kushwaha et al., 2016), but it must pass through the plasma membrane of the endodermis cell before entering the stele of roots for long-distance transport (Verbruggen et al., 2009; Xu and Wang, 2013). The Cd binding by cell walls of rhizodermis and cortex tissues may serve as the first barrier to reduce the active Cd^2+^ and lessen the damage to root cells and/or the amount of Cd^2+^ transported into the stele (Nishizono et al., 1989; Verkleij and Schat, 1990). In this study, Kentucky bluegrass showed the strongest Cd fluorescence in the stele and less fluorescence in epidermic and cortical tissues (**Figure 1D**), indicating that the most Cd absorbed by roots was transported into the stele for further root-to-shoot translocation, as shown by the higher Cd concentration accumulated in leaves of Kentucky bluegrass (**Figure 4**).

Mesophyll is the critical functional tissues in the leaf for photosynthesis (Ebbs et al., 2009). The weaker Cd fluorescence in mesophyll tissues of tall fescue indicated that the photosynthetic apparatus in tall fescue might suffer less Cd toxicity than that in Kentucky bluegrass.

Qi et al. (2014) reported that Rhodes grass (*Chloris gayana*) could excrete salt out of the cuticle surface via salt glands. In this study, we observed strong Cd fluorescence in the cuticle layer of the leaf epidermis, which indicated that tall fescue and Kentucky bluegrass could excrete Cd out of the leaf tissues to avoid Cd damages. More uniform and stronger Cd fluorescence in the cuticle layer of tall fescue leaves indicated the higher capacity of Cd excretion. The mechanisms of how leaves of these two turfgrass species to excrete Cd to the leaf surface are unclear, which deserves further investigation.

## Conclusion

There was no difference in root Cd accumulations between tall fescue and Kentucky bluegrass. Kentucky bluegrass transported more Cd into the stele and translocated from roots to shoots, resulting in significantly higher Cd concentrations in leaves, compared with tall fescue. The less Cd transported into the stele in roots and more Cd excreted to the cuticle layer in the leaf of tall fescue could contribute to its high level of Cd hypertolerance.

## Author Contributions

PX and ZW conceived and designed the experiment. QD and PX conducted the experiment. QD and ZW analyzed data and wrote the manuscript. The authors read and approved the paper.

## Conflict of Interest Statement

The authors declare that the research was conducted in the absence of any commercial or financial relationships that could be construed as a potential conflict of interest.
