# Supplementary material for: Differential Cadmium Distribution and Translocation in Roots and Shoots Related to Hyper-Tolerance between Tall Fescue and Kentucky Bluegrass
Source: Front Plant Sci. 2017 Feb 3;8:113. doi: 10.3389/fpls.2017.00113 (PMC5289992; doi:10.3389/fpls.2017.00113)
Supplement: Supplementary file 1 [file Data_Sheet_1.docx]

Supplementary photos from histochemical method (from experiment I)

Fig. 1 Cd distribution in the root tissues of tall fescue (a, b, c) and Kentucky bluegrass (d, e, f). a: tall fescue control; b, c: tall fescue Cd treatment under 0.3 mM Cd^2+^ for 21 d; d: Kentucky bluegrass control; e, f: Kentucky bluegrass Cd treatment under 0.3 mM Cd^2+^ for 21 d. Bars on photos is 10 μm length. R- rhizodermis; C- cortex; E- endodermis; Pe- pericycle; Ph- phloem; X- xylem.

The specific pink-orange color represented the Cd dithizone stain for Cd locations according to Seregin and Kozhevnokova (2011). Tall fescue showed more Cd distribution in the cortex (b) and less Cd in the stele (c) when compared to Kentucky bluegrass (e and f).


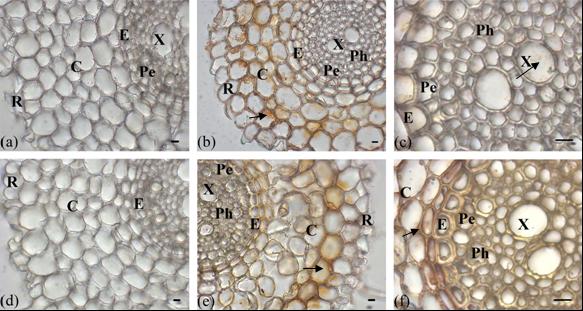


Fig. 2 Cd distribution in the leave tissues of tall fescue (a, b, c) and Kentucky bluegrass (d, e, f). a: tall fescue control; b, c: tall fescue Cd treatment under 0.3 mM Cd^2+^ for 21 d; d: Kentucky bluegrass control; e, f: Kentucky bluegrass Cd treatment under 0.3 mM Cd^2+^ for 21 d. Bars on photos is 10 μm length. uE- upper epidermis; lE- lower epidermis; M- mesophyll; Vb- vascular bundle; Ph- phloem; X- xylem.

The specific pink-orange color represented the Cd dithizone stain for Cd locations according to Seregin and Kozhevnokova (2011). Vascular bundle showed more Cd colors both in tall fescue (c) and Kentucky bluegrass (f).


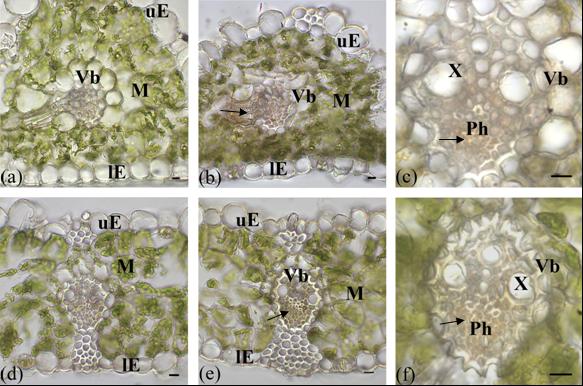


Supplementary Cd concentrations measured from roots and shoots

Experiment I

Table 1 The concentration and accumulation of Cd in shoots and roots after 21 d of 0.3 mM Cd^2+^ treatments in hydroponic system. The data are presented by means ± standard errors of four replications. Different letters represent the significant differences between Kentucky bluegrass and tall fescue at LSD 0.05.

| Turfgrass  species | Cd concentration  (mg kg^-1^ DW) | | Translocation factor (S/R) |
| --- | --- | --- | --- |
|  | Shoots | Roots |  |
| Kentucky bluegrass | 355.8±20.5 a | 2905.0±247.1 a | 0.123±0.016 a |
| Tall fescue | 273.3±15.8 b | 2817.2±206.6 a | 0.097±0.003 b |
